# Transcutaneous vagus nerve stimulation reduces total striatal GABA+ content, increases DLPFC Glu content, and facilitates early-phase motor learning

**DOI:** 10.3389/fnhum.2026.1731345

**Published:** 2026-02-18

**Authors:** Kana Matsumura, Hiroyuki Matsuta, Ryushin Kawasoe, Tomoyuki Fumuro, Kojiro Matsushita, Nobuhiro Hata, Yoshiki Asayama, Tsuyoshi Shimomura, Minoru Fujiki, Hisato Sugata

**Affiliations:** 1 Graduate School of Welfare and Health Science, Oita University, Oita, Japan; 2 Hospital Informatics Center, Oita University Hospital, Oita, Japan; 3 Department of Neurosurgery, School of Medicine, Oita University, Oita, Japan; 4 Division of Health Sciences, Graduate School of Medicine, Osaka University, Osaka, Japan; 5 Faculty of Medicine, Department of Advanced Medical Sciences, Oita University, Oita, Japan; 6 Department of Mechanical Engineering, Gifu University, Gifu, Japan; 7 Faculty of Medicine, Department of Radiology, Oita University, Oita, Japan; 8 Faculty of Welfare and Health Science, Oita University, Oita, Japan; 9 Graduate School of Medicine, Oita University, Oita, Japan

**Keywords:** gamma-aminobutyric acid, glutamate, proton magnetic resonance spectroscopy, motor learning, neuromodulation, tVNS

## Abstract

**Objectives:**

Transcutaneous vagus nerve stimulation (tVNS) has emerged as a promising non-invasive technique for modulating neuroplasticity. Previous studies have suggested that changes in regional brain GABA+ signaling contribute to these effects, but empirical neurophysiological evidence remains limited.

**Materials and methods:**

We investigated the neurophysiological and behavioral effects of tVNS (200-μs pulses at 20 Hz, alternating 30 s ON–1 s OFF cycles, 30 min total duration) in healthy adults using two experimental paradigms. In Experiment 1, GABA+ and Glutamate (Glu) levels were measured in the left striatum (STR), dorsolateral prefrontal cortex (DLPFC), and sensorimotor cortex (SM) of 34 participants by proton magnetic resonance spectroscopy (^1^H-MRS), before and after ipsilateral tVNS. In Experiment 2, 27 participants performed a right-hand force-control motor learning task before, during, and after tVNS.

**Results:**

Administration of tVNS significantly reduced GABA+ levels in the left STR (*p* < 0.05), increased Glu levels in the DLPFC (*p* < 0.05), and significantly improved motor task performance compared to the sham group at 10 min after stimulus onset (*p* < 0.05).

**Conclusion:**

tVNS (1) reduced striatal GABA+ levels and increased DLPFC Glu in healthy adults, and (2) facilitated early-phase motor learning. These findings support the use of tVNS as a noninvasive intervention that enhances motor learning in neurorehabilitation and the treatment of motor disorders.

## 1 Introduction

Invasive vagus nerve stimulation (iVNS) has been approved by the U. S. Food and Drug Administration as a treatment for epilepsies and depression as well as for stroke rehabilitation, and numerous clinical studies and reviews have confirmed the efficacy of this intervention (Fisher et al., 2021; González et al., 2019). For instance, a comprehensive review reported that iVNS reduced seizure frequency by 50–100% in 45–65% of patients (Toffa et al., 2020). However, iVNS requires surgical electrode implantation, and thus carries the risks of surgical side effects. Implantation surgery is also costly and not widely accessible, especially in developing countries. Conversely, transcutaneous vagus nerve stimulation (tVNS) provides a non-invasive, lower cost, and more accessible alterative (Kim et al., 2022; Redgrave et al., 2018; Tan et al., 2023), and in the two decades since it was first introduced has been reported to enhance social behavior in epilepsy patients (Oehrn et al., 2022), aid in post-stroke motor recovery (Capone et al., 2017; Shi et al., 2023), improve both motor and non-motor symptoms of Parkinson’s disease (Shan et al., 2025), provide symptomatic relief from depression (Fang et al., 2016; Kong et al., 2018) and post-traumatic stress disorder (PTSD) (Lamb et al., 2017), and even suppress postoperative pain following total knee arthroplasty (Aoyagi et al., 2025).

The primary acute outcome of tVNS is a shift in autonomic nervous system function toward parasympathetic dominance (Badran et al., 2018; Briand et al., 2020), but the downstream neurological changes underlying many of the reported therapeutic effects remain unknown. Recent evidence suggests that tVNS promotes neuroplasticity in midbrain and corticobasal pathways, potentially by modulating neurotransmitter metabolism and signaling (Beste et al., 2016; Briand et al., 2020). For instance, tVNS via the left cymba conchae has been shown to activate the nucleus tractus solitarii and the locus coeruleus, leading to increased noradrenergic neurotransmission (Ay et al., 2015; Frangos et al., 2015; Hansen, 2019; Ruffoli et al., 2011; Teckentrup et al., 2021). Another potential action mechanism is altered gamma-aminobutyric acid (GABA+) signaling (Colzato et al., 2018; Jongkees et al., 2018; Keute et al., 2018). GABA+ is the primary inhibitory neurotransmitter in the brain and several studies have reported the iVNS can increase neural GABA levels (Ben-Menachem et al., 1995). Also, long-term iVNS was reported to increase hippocampal GABA receptor density in the hippocampus of patients with drug-resistant epilepsy (Marrosu et al., 2003). Thus, tVNS may exert therapeutic effects by modulating GABAergic signaling. In fact, this notion is indirectly supported by neuroimaging studies. Multiple studies have reported an inverse correlation between GABA+ levels and blood oxygen level-dependent (BOLD) signals (Jung et al., 2025; Levar et al., 2017; Northoff et al., 2007). For example, Stagg et al. (2011) reported that decreases in GABA+ levels were associated with elevated BOLD signals in the sensorimotor cortex (SM), and Dolfen et al. (2021). reported that increased striatal GABA+ was associated with reduced BOLD signals. Furthermore, Frangos and Komisaruk reported that tVNS increased BOLD signals in the basal ganglia, including the STR (Frangos and Komisaruk, 2017), suggesting a reduction in striatal GABA+ levels. Although there is growing interest in the neuromodulatory effects of tVNS for therapeutic intervention, the underlying mechanisms must be clarified. In particular, there is little direct neurophysiological evidence for modulation of GABAergic signaling by tVNS (Keute et al., 2018; Keute et al., 2021).

One especially promising application of tVNS is in motor rehabilitation, a therapeutic process that depends strongly on motor learning. Motor learning refers to the process by which individuals acquire new skills or refine previously learned skills, allowing them to adapt more effectively to an ever-changing environment (Galea et al., 2011; Halsband and Lange, 2006). This phenomenon involves structural and functional changes in several brain regions, such as STR (Gann et al., 2021), SM (Jin et al., 2019; Fitzroy et al., 2021; Li et al., 2022), and the dorsolateral prefrontal cortex (DLPFC) (Jin et al., 2019; Gann et al., 2021), and is associated with various regional changes in neurotransmitter signaling. Inhibitory control of corticobasal circuits mediated by GABAergic signaling is particularly important for motor learning (Li et al., 2022), and several studies have reported that tVNS can enhance motor sequence learning (Jongkees et al., 2018) and promote memory consolidation skills in healthy individuals (Burger et al., 2016). Reduced GABAergic activity is further implicated in these effects as tVNS has been shown to enhance BOLD activity, which in turn is negatively correlated with GABAergic transmission. However, several studies have reported that tVNS can also increase regional GABA+ (Jongkees et al., 2018; Keute et al., 2018; Beste et al., 2016; Colzato and Beste, 2020). Therefore, additional studies investigating the effects of tVNS on both GABA+-associated neuroimaging signals and motor learning are required.

In addition to inhibitory GABAergic transmission, excitatory glutamatergic neurotransmission also plays a role in learning. Glutamate (Glu) is the principal excitatory neurotransmitter in the human brain. Motor learning is facilitated by the modulation of striatal GABA and cortical Glu (Reinhold, 2025; Wittenberg, 2017). However, despite growing interest in the neuromodulatory effects of tVNS, evidence is scarce on the effects of tVNS on regional Glu levels in motor-related brain regions.

We speculated that tVNS promotes motor task performance by modulating GABA+ and Glu levels or signaling within STR, DLPFC, or SM circuits. To test this hypothesis, we measured the effects of tVNS on GABA+ and Glu levels in the STR, DLPFC, and SM using proton magnetic resonance spectroscopy (^1^H-MRS) (Experiment 1), as all of these regions are implicated in motor learning, and further examined the effects of tVNS on the acquisition of a motor learning task (Experiment 2).

## 2 Materials and methods

### 2.1 Ethics statement

All experimental protocols were approved by the Ethics Review Board of the Oita University Faculty of Welfare and Health Science (No. F240033) and conducted in accordance with the tenets of the Declaration of Helsinki. The study purpose and possible consequences were fully explained prior to experiments, and informed consent was obtained from all participants.

### 2.2 Transcutaneous vagus nerve stimulation

**Figure 1:**
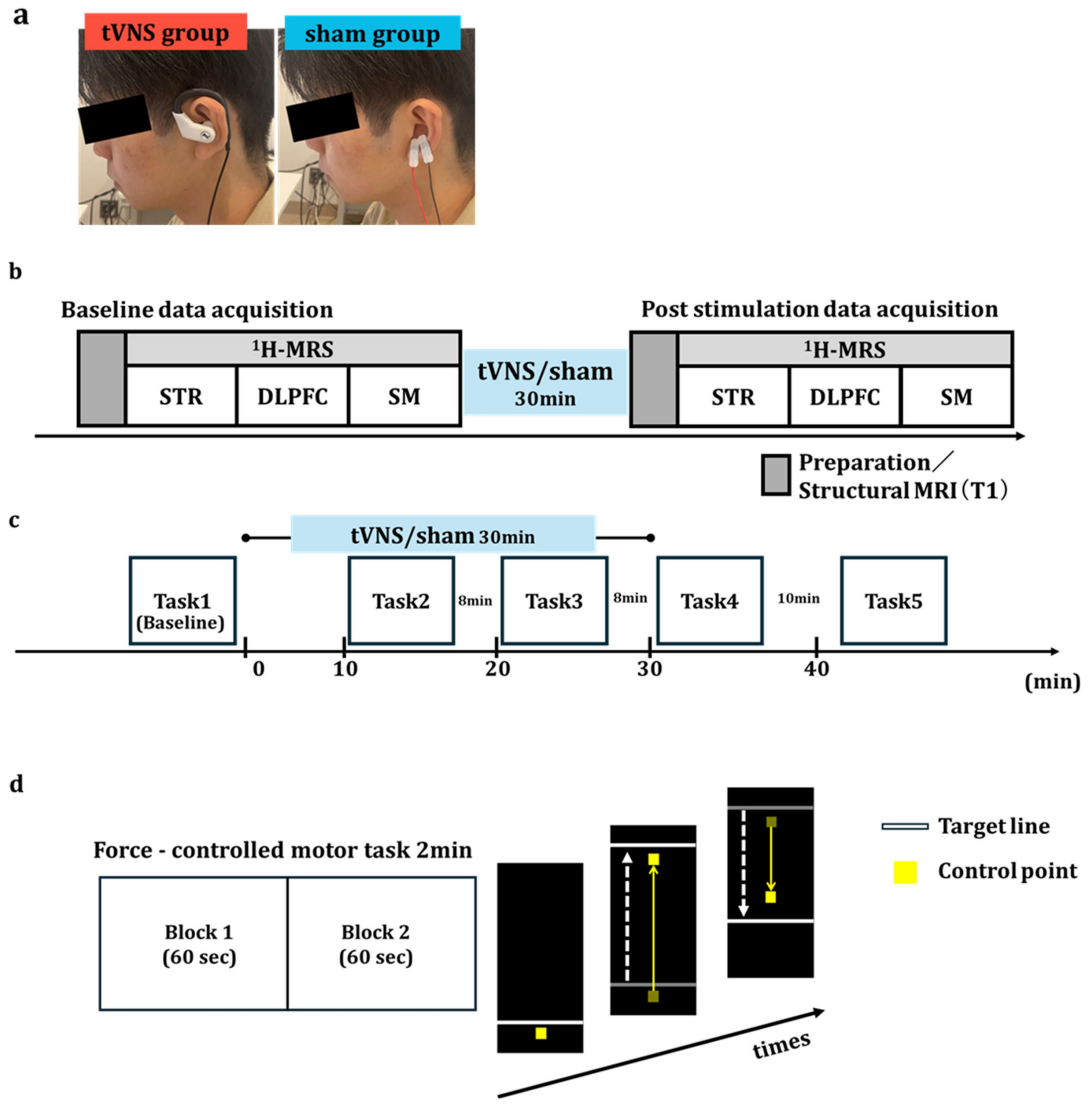
Experimental design. **(a)** Stimulation sites for transcutaneous vagus nerve stimulation (tVNS) and sham groups. The tVNS group was stimulated at the left cymba conchae (left panel), while the sham group was stimulated at the left earlobe (right panel). **(b)** Scheme for Experiment 1. Participants lie in an MRI scanner to measure the baseline GABA^+^ concentration in striatum (STR), dorsolateral prefrontal cortex (DLPFC), and sensorimotor cortex (SM). After that, they received either 30 min of tVNS or sham stimulation in a separate room. Following stimulation, GABA^+^ concentration was remeasured in the same region. **(c)** Scheme for Experiment 2. A 2-min force-controlled task was performed before stimulation (Task 1), 10 min after stimulation onset (Task 2), 20 min after stimulation onset (Task 3), immediately after the end of the stimulation (Task 4), and 12 min after the end of the stimulation (Task 5). **(d)** Motor task protocol. Each trial consisted of two 60-s blocks and lasted 2 min. Participants were instructed to adjust the pinch force using their right thumb and index finger to quickly and accurately match a randomly moving target line.

In both Experiments 1 and 2, the vagus nerve was stimulated noninvasively using a specialized tVNS device (Soterix Medical, Inc., USA). In the tVNS group, the stimulation electrode was placed on the left cymba conchae to target the auricular branch of the left vagus nerve, while in the corresponding sham group, electrodes were positioned on the left earlobe, an area not innervated by the vagus nerve and thus not expected to induce vagal activation (Figure 1a) (Ellrich, 2019; Peuker and Filler, 2002; Yakunina et al., 2017). The tVNS device was set to deliver electrical stimulation with a pulse width of 200 μs at 20 Hz, alternating between 30 s ON and 1 s OFF, for a total duration of 30 min. Previous studies have shown that a stimulation intensity around 1.0 mA can induced cortical plasticity (Borland et al., 2016; Carroll et al., 2024; Morrison et al., 2019; Pruitt et al., 2021), so 1.0 mA was chosen for both tVNS and sham stimulation. To assess the potential side effects of tVNS, all participants completed visual analog scales (VASs) rating pain, itchiness, and discomfort after the experiments.

### 2.3 Experiment 1: Effects of tVNS on regional GABA+ and Glu levels measured by ^1^H-MRS

#### 2.3.1 Participants

Thirty-four healthy adults (17 females, 17 males, mean age = 22.7 ± 2.8 years, range = 19–29 years) were recruited for Experiment 1 to investigate the effects of tVNS on regional brain GABA+. Inclusion criteria were (i) right-handed and (ii) 18 to 29 years of age, while exclusion criteria were (i) claustrophobia, (ii) implanted metal objects, (iii) history of psychiatric disorders, and (iv) current use of psychotropic medication. All participants were right-handed as determined by the Edinburgh Handedness Inventory (EHI) (Oldfield, 1971). After enrolment, participants were randomly assigned to the tVNS group (*n* = 17) or the sham group (*n* = 17).

#### 2.3.2 Experimental design

After screening for implanted metallic materials, the participants were positioned in the MRI scanner and baseline GABA+ levels measured using proton magnetic resonance spectroscopy (^1^H-MRS). Following baseline measurements, participants of the tVNS and sham groups received the pre-allocated treatment (tVNS applied to the left cymba conchae or sham stimulation to the left ear lobe for 30 min) in a separate room. Post-stimulation GABA+ levels were then measured during a second MRI session (Figure 1b).

#### 2.3.3 ^1^H-MRS and structural MRI acquisition

Magnetic resonance spectroscopic and structural imaging data were acquired on a 3-T MEGNEATOM Skyra Fit scanner equipped with a 32-channel head coil. All ^1^H-MRS and MRI measurements were performed by a neurosurgeon (author HM) with extensive experience in ^1^H-MRS techniques. T1-weighted images were obtained in the axial, coronal, and sagittal planes to define the STR, DLPFC, and SM as volumes of interest (VOIs). The total volumes for STR, DLPFC, and SM were 30 × 25 × 25 mm^3^, 30 × 25 × 25 mm^3^, and 25 × 25 × 25 mm^3^, respectively. MEGA-PRESS-edited GABA+ spectra (Mescher et al., 1998; Edden and Barker, 2007; Matsuta et al., 2022) were acquired from each VOI using the following parameters: repetition time (TR) = 3 s; echo time (TE) = 68 ms; editing pulse applied either at 3.0 ppm (ON) or 6.4 ppm (OFF); segments of 2048 data points each for ON and OFF acquisitions; spectral bandwidth of 2 kHz interleaved 64 times. In addition, eight dummy pulse scans were performed for signal normalization. In total, 136 scans were acquired from each participant, and total acquisition time was 408 s. Water suppression was performed using the VAPOR sequence. Prior to the ^1^H-MRS scan, all VOIs underwent manual shimming. Manual adjustments were made to achieve an FWHM ≤ 20 Hz for the STR, and ≤15 Hz for the DLPFC and SM.

#### 2.3.4 ^1^H-MRS analysis

^1^H-MRS data were imported into the LCmodel (version 6.3-1 N) spectral analysis software program (http://lcmodel.ca/lcmodel.shtml), corrected for frequency and phase drift in 64 edit-ONs and OFFs, and then Gaussian filtered (2 Hz) and Fourier transformed prior to within- and between-group comparisons. Based on the water reference scan, we performed eddy current correction and water scaling. The GABA+ signal, a high-molecular-weight spectral peak containing GABA and other metabolites, was quantified from the spectrum edited using the LCmodel. Because the absolute GABA+ concentration is difficult to quantify, this paper reported the ratio of GABA+ to the simultaneously acquired NAA peak as the “GABA+ level.” ^1^H-MRS voxels were then coregistered with the structural MRI image of each participant and segmented to determine the fractions of gray matter (GM), white matter (WM), and CSF using GANNET software (version 3.1.5) (https://markmikkelsen.github.io/Gannet-docs/). Regional differences in GABA+ concentrations are negligible in CSF but approximately twice as large in GM than WM, so tissue correction was applied.

#### 2.3.5 VOI analysis

To ensure that ^1^H-MRS voxels were consistently located in the same brain regions between participants, groups, and pre- and poststimulation scans, voxel masks were normalized to Montreal Neurological Institute space using SPM25 (https://www.fil.ion.ucl.ac.uk/spm/). The spatial overlap between pre- and poststimulation VOIs was then visualized using MRIcroGL (https://www.nitrc.org/projects/mricrogl).

#### 2.3.6 Statistical analysis

Regional GABA+ levels were compared between tVNS and sham groups by independent samples Student’s t-tests, while the VOI overlap between pre- and post-scans in tVNS and sham groups was compared by paired Student’s t-tests to confirm the consistency of the VOI location.

### 2.4 Experiment 2: Effects of tVNS on motor learning

#### 2.4.1 Participants

Twenty-seven right-handed healthy participants (14 males, 13 females, mean age = 20.9 ± 0.9 years, range = 19–23 years) were recruited for Experiment 2 to investigate the effect of tVNS on motor learning. Inclusion criteria were (i) right-handed and (ii) 18 to 29 years of age, while exclusion criteria were (i) psychiatric diagnoses and (ii) current treatment with psychotropic agents. All participants were right-handed as determined by the EHI (Oldfield, 1971). Again, participants were randomly assigned to a tVNS group (*n* = 14) or sham group (*n* = 13). One of the participants recruited in Experiment 1 also participated in Experiment 2. Considering the lasting effects of tVNS, a 4-month interval was maintained for this participant between the two experiments.

#### 2.4.2 Experimental design and motor learning task

In Experiment 2, we investigated the effects of tVNS on motor learning using a time-series design. Participants completed a force-control task at five different time points: before tVNS or sham stimulation (Task 1), 10 min (Task 2) and 20 min (Task 3) after stimulus onset, immediately after stimulation (Task 4), and 12 min post-stimulation (Task 5). As in Experiment 1, 30 min of tVNS or sham stimulation was administered during the session, and motor performance was evaluated repeatedly to capture the learning process across time (Figure 1c).

The force-control motor task was conducted as described in a previous study (Floyer-Lea and Matthews, 2004). Briefly, each trial consisted of two, 60-s blocks and lasted 2 min. During the task, participants used a pressure sensor (Interlink Electronics Inc., USA) operated with their right thumb and index finger to control the vertical movement of a yellow point on a computer screen (Figure 1d). By adjusting the pinch force, they attempted to align the yellow point with a white target line that moved in a pseudorandom pattern across five force levels (0.5, 1.0, 1.5, 2.0, and 2.5 N). For each force level, amplitude rose from 0 N to the target value and back to 0 N within 6 s. We calculated the mean absolute error (N) between the target line and the actual force output separately for each block to quantify motor performance (Blocks 1 to 8). To evaluate the effect of stimulation on motor learning, group comparisons were performed at each corresponding block using the unpaired Student’s t-test.

## 3 Results

### 3.1 Experiment 1: Effects of tVNS on regional GABA+ levels

#### 3.1.1 Side effects of tVNS and sham stimulation

Five of 17 participants reported minor itchiness (mean VAS score, 3.3 ± 2.6) and three minor discomfort (mean 2.3 ± 0.9) during tVNS, but none reported pain. Two of 17 participants in the sham group also reported minor itchiness (mean = 3.4 ± 1.3), whereas none reported pain or discomfort.

#### 3.1.2 Comparisons of regional GABA+ levels and Glu levels before and after tVNS

**Figure 2:**
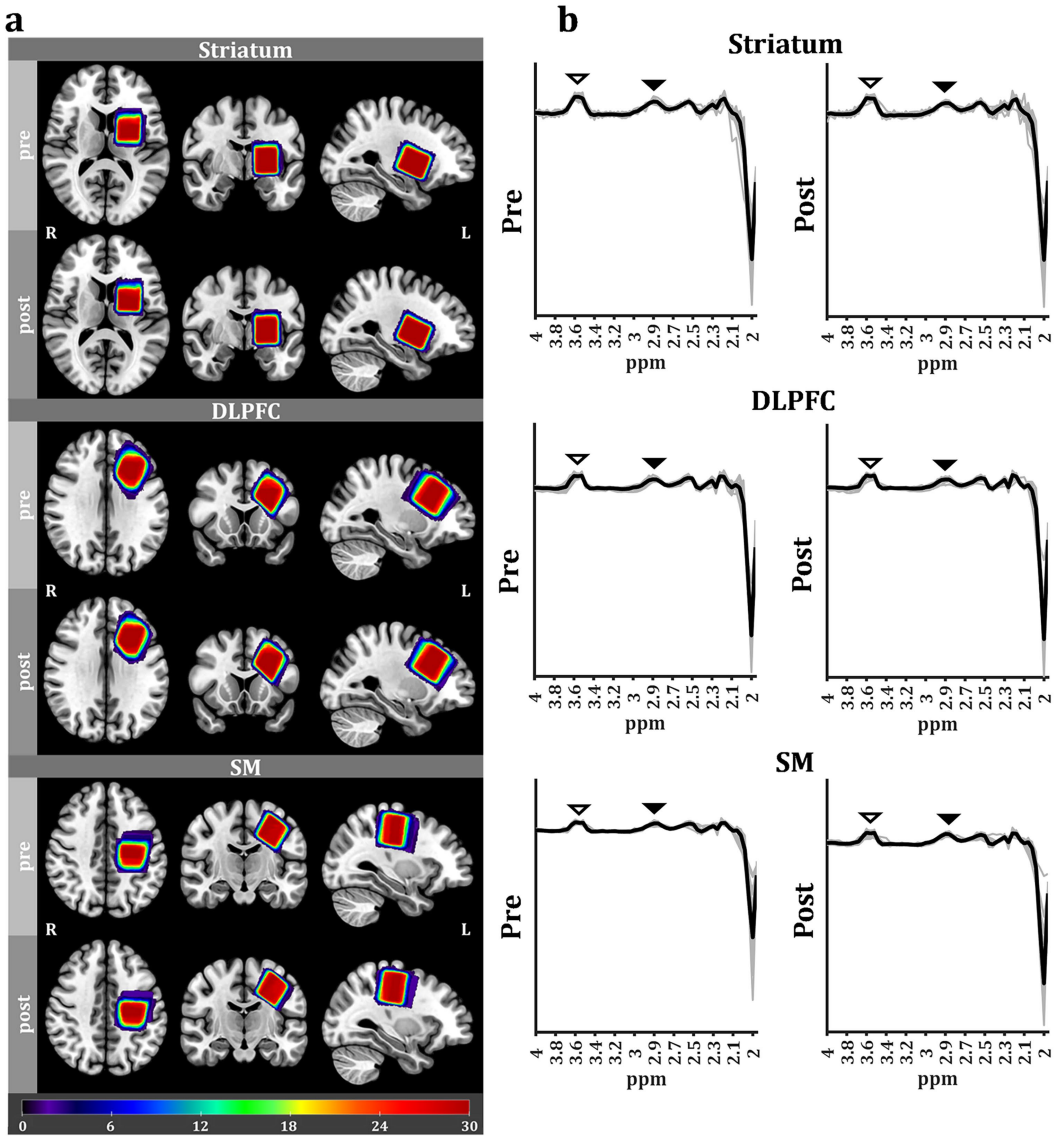
VOI analysis and the ^1^H-MRS-GABA+ spectrum. **(a)** VOI analysis. Heatmaps representing the spatial overlap across participants for STR, DLPFC, and SM ^1^H-MRS voxels (pre; upper, post: bottom). Color bars represent the number of overlapping voxels. Heatmaps are overlaid over the mean structural image across all participants. The high degree of spatial overlap indicates a high consistency in voxel placement across time points and individuals. **(b)** Pooled pre- and poststimulation ^1^H-MRS-GABA+ spectrum data in each brain region. Black solid lines indicate averaged ^1^H-MRS spectrum data across all participants, and gray solid lines indicate individual-participant ^1^H-MRS spectrum data. Black arrowheads indicate the GABA+ peaks; white arrowheads indicate the Glu peaks.

No adverse effects of ^1^H-MRS were reported, and GABA+-edited ^1^H-MRS data were obtained from all 34 participants in Experiment 1. However, 4 participants (2 in each group) were excluded due to data corruption during acquisition. As a result, data from 15 tVNS group and 15 sham group participants were included in the final analyses (Table 1, upper). Before analyzing GABA+ levels, we compared the VOI overlap for each target region between treatment groups, and found no significant differences (Figure 2a; Table 2). Thus, differences in GABA+ were not influenced by inaccuracies in setting VOI boundaries.

**Table 1:** Demographic characteristics of the participants.

Experiment 1	tVNS group	Sham group	*p* value
Gender (male/female)	8/7	8/7	1
Age (years; mean ± SD)	21.2 ± 1.05	23.53 ± 3.14	0.072
EHI score (mean ± SD)	96.41 ± 6.37	93.04 ± 12.77	0.810

Experiment 2	tVNS group	Sham group	*p* value
Gender (male/female)	8/6	6/6	0.716
Age (years; mean ± SD)	21.14 ± 1.06	20.58 ± 0.76	0.154
EHI score (mean ± SD)	96.16 ± 6.52	86.45 ± 13.70	0.057

EHI, Edinburgh handedness inventory.

**Table 2:** Regional volume of interest overlap between measurement times.

Brain regions	tVNS group	Sham group	*t* value	*p* value
STR (%, mean ± SD)	83.35 ± 4.15	87.36 ± 5.56	−1.751	0.102
DLPFC (%, mean ± SD)	81.03 ± 4.14	79.12 ± 8.59	0.829	0.421
SM (%, mean ± SD)	83.12 ± 7.43	83.89 ± 7.42	−0.276	0.786

STR, striatum; DLPFC, dorsolateral prefrontal cortex, SM, sensorimotor cortex.

**Figure 3:**
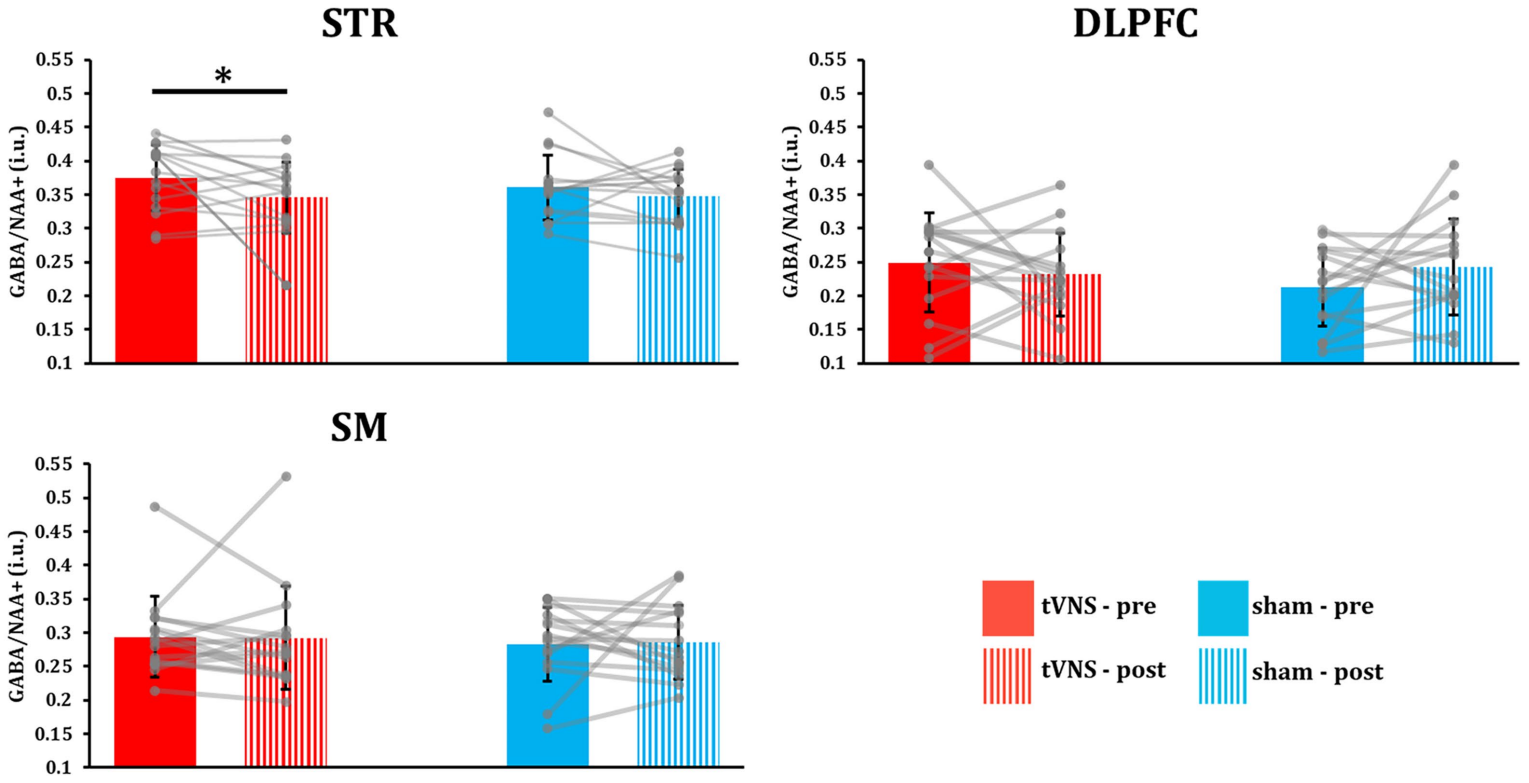
tVNS reduced total GABA+ concentration in the striatum. STR: Striatum; DLPFC: Dorsolateral prefrontal cortex; SM: Sensorimotor cortex. Striatal GABA+ significantly decreased following tVNS but not sham stimulation (*p = 0.041). No significant changes were observed in the DLPFC or SM. Error bars indicate ±standard deviation.

Regional ^1^H-MRS-GABA+ spectra following tVNS or sham stimulation were then compared to corresponding baselines (Figure 2b). Striatal total GABA+ level was significantly lower following tVNS compared to baseline (*p* = 0.041, *t-value* = −1.861, *Cohen’s d* = −0.481) (Figure 3), while no significant changes were observed in the DLPFC (*p* = 0.222, *t-*value = −0.786, Cohen’s *d* = −0.203) and SM (*p* = 0.465, *t-*value = −0.090, Cohen’s *d* = −0.023), although there was a numeric decrease in the DLPFC. Alternatively, no significant changes were observed in the STR (*p* = 0.180, *t-*value = −0.943, Cohen’s *d* = −0.066), DLPFC (*p* = 0.890, *t-*value = 1.286, Cohen’s *d* = 0.325), and SM (*p* = 0.562, *t-*value = 0.160, Cohen’s *d* = 0.058) following sham stimulation, although a numeric increase was observed in the DLPFC.

**Figure 4:**
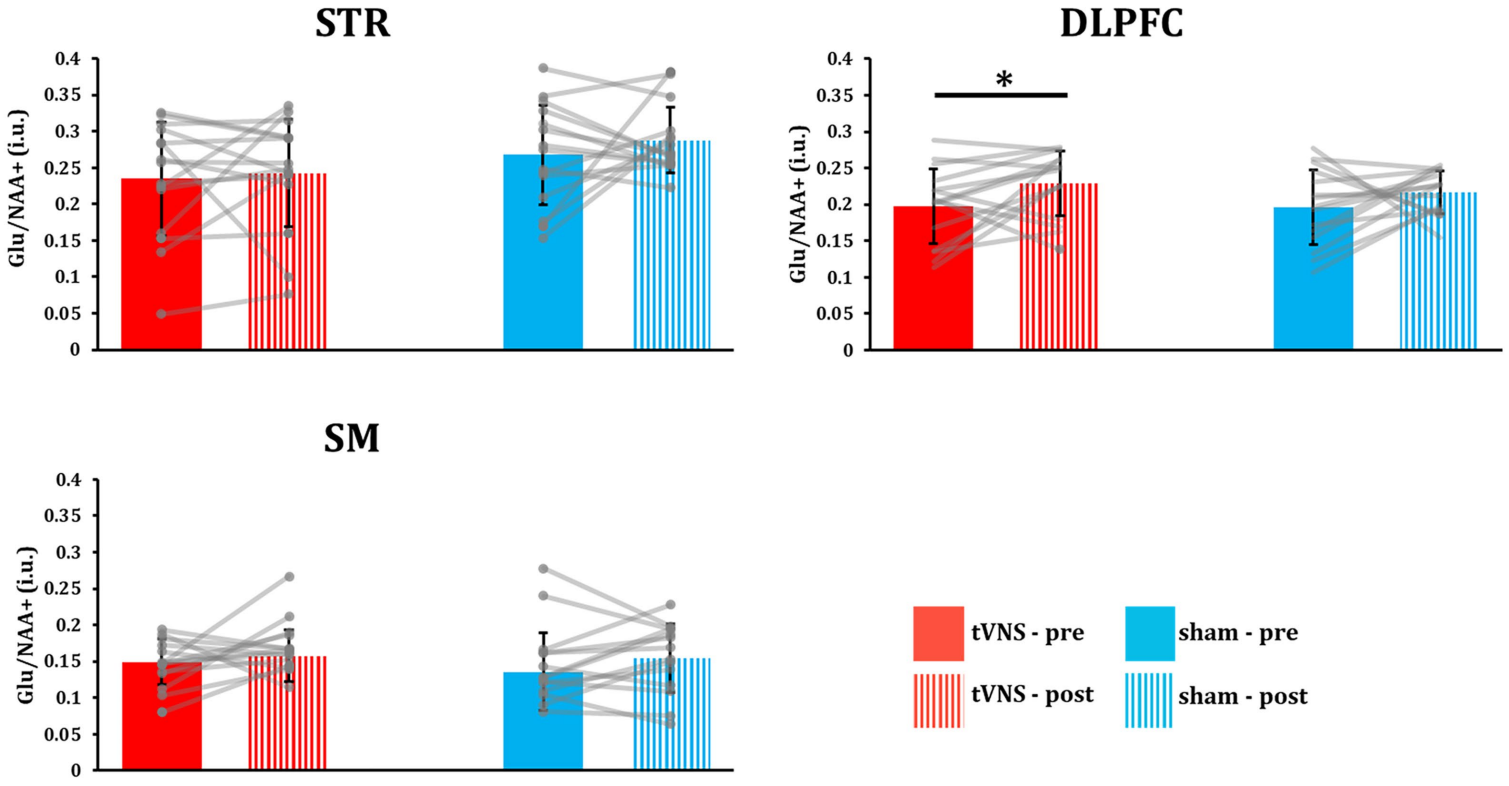
TVNS increased total Glu concentration in the DLPFC. STR: triatum; DLPFC: orsolateral prefrontal cortex; SM: ensorimotor cortex. DLPFC Glu significantly increased following tVNS but not sham stimulation (*p < 0.05). No significant changes were observed in the STR or SM. Error bars indicate the ±standard deviation.

Regional ^1^H-MRS-Glu spectra following tVNS or sham stimulation were then compared with the corresponding baselines (Figure 2b). DLPFC total Glu levels significantly increased following tVNS (*p* = 0.030, *t-*value = 2.046, Cohen’s *d* = 0.528; Figure 4), while no significant changes were observed in the STR (*p* = 0.353, *t-*value = 0.386, Cohen’s *d* = 0.100) and SM (*p* = 0.326, *t-*value = 0.462, Cohen’s *d* = 0.119). Conversely, no significant changes were observed in the STR (*p* = 0.182, *t-*value = 0.940, Cohen’s *d* = 0.243), DLPFC (*p* = 0.129, *t-*value = 1.181, Cohen’s *d* = 0.305), and SM (*p* = 0.116, *t-*value = 1.248, Cohen’s *d* = 0.322) following sham stimulation.

### 3.2 Experiment 2: Effects of tVNS on motor task performance

#### 3.2.1 Side effects of tVNS and sham stimulation

Two participants reported mild pain (mean VAS score 2.6 ± 1.0), four reported moderate itchiness (mean 4.3 ± 1.5), and four experienced minor discomfort (mean 1.5 ± 0.8) following tVNS. Two participants reported mild pain (mean 2.3 ± 0.8), one reported itchiness (1.6), and one felt discomfort (2.3) following sham stimulation.

#### 3.2.2 Task performance improvement by tVNS

One participant who could not adequately manipulate the force sensor in the force-control motor task was excluded from the analysis. Thus, the final sample sizes were 14 for the tVNS group and 12 for the sham group (Table 1, lower). The mean error values in each block were defined as learning values and compared across blocks. Mean error values were significantly lower following 10 min of tVNS compared to 10 min of sham stimulation (*p* = 0.023, *t-value* = −2.635, *Cohen’s d* = −1.037). Alternatively, performance levels did not differ after 20 min of tVNS, immediately after 30 min of tVNS, or 12 min after 30 min of tVNS compared to the sham stimulation condition.

## 4 Discussion

### 4.1 Effects of tVNS on GABA+ and Glu levels

In Experiment 1, we found that tVNS reduced total GABA+ content in the left STR, although no significant changes were observed in the DLPFC or SM. In addition, tVNS significantly increased left DLPFC Glu levels, although no significant changes were observed in the STR or SM. A previous study (Li et al., 2019) similarly reported that tVNS reduced GABA+ levels in the anterior cingulate cortex of a patient with treatment-resistant depression. Thus, tVNS may not simply enhance regional GABA+ contents as reported in multiple previous studies, but may also reduce levels, at least in some regions, an effect that could in turn facilitate neuroplasticity through disinhibition of various motor-related circuits. Indeed, we observed enhanced learning of a force-control motor task during tVNS. While this effect appeared early and transient (only after 10 min of stimulation), task performance was also rapidly reaching a plateau. Therefore, additional studies are needed to examine if tVNS enhances motor learning across phases and task conditions, such as during more difficult tasks with no rapid ceiling effect (or floor effect on error rate as measured in the present study). If so, tVNS could be a broadly useful adjunct treatment for recovery of motor function.

The striatum contains abundant GABAergic interneurons and short projection neurons that facilitate motor learning by modulating cortical plasticity through direct and indirect pathways (Cataldi et al., 2022). The present findings suggest that a decrease in striatal GABA+ and ensuing disinhibition may have contributed to faster learning of the force-control task via effects on corticostriatal plasticity. Farrand et al. (2017). reported that iVNS increased the expression of multiple neurotransmitters in the STR and concomitantly mitigated motor deficits in a rat model of Parkinson’s disease, possibly by enhancing striatal output through disinhibition and strengthening of network connectivity as a previous reported that tVNS enhanced functional connectivity within the striatum-prefrontal cortex network (Wang et al., 2018). Additionally, tVNS increased BOLD signals in the STR (Frangos and Komisaruk, 2017) and there is a negative correlation between GABA+ levels and BOLD signals (Jung et al., 2025; Levar et al., 2017; Northoff et al., 2007; Stagg et al., 2011; Dolfen et al., 2021), further supporting a tVNS-induced decrease in striatal GABA+ levels.

Although no significant differences were observed, tVNS also numerically reduced GABA+ in the DLPFC, whereas the sham group showed an increase, suggesting enhanced activation of STR–DLPFC circuits by tVNS. Capone et al. (2015). reported that left-sided tVNS increased short-interval intracortical inhibition, which is reflective of GABA-A inhibitory circuit activity, in the right SM but not the left SM, so enhanced GABAergic inhibitory activity in the right (or contralateral) SM cannot be ruled out. Alternatively, it is also possible that the absence of observable GABA+ modulation in the SM may be due to the delay in ^1^H-MRS acquisition (~40 min after tVNS). In contrast to the GABA+ findings, tVNS significantly increased DLPFC Glu levels.

One possible mechanism underlying this effect is the activation by tVNS of ascending neuromodulatory pathways. Stimulation of the auricular branch of the vagus nerve can activate the nucleus tractus solitary and the locus coeruleus, increasing noradrenergic input to prefrontal regions (Briand et al., 2020). Noradrenergic signaling facilitates glutamatergic synaptic transmission and increases cortical excitability, which may preferentially enhance excitatory activity in the DLPFC following tVNS.

Together with the observed reduction in striatal GABA+, these findings suggest that tVNS induces region-specific modulation of neurotransmission within cortico-basal networks, characterized by disinhibition in the STR and enhanced excitatory drive in prefrontal control regions. Such coordinated modulation may support efficient motor learning by facilitating both motor execution and higher-order cognitive control processes, consistent with the early and transient learning effects observed in the present study.

### 4.2 Acceleration of motor learning by tVNS

Administration of tVNS for 10 min enhanced motor learning, although not maximum performance, suggesting a specific influence on early-phase motor learning. However, as discussed, longer term effects during stimulation may have been obscured by floor effect on the error rate, so further studies are warranted to examine the efficacy for improving performance on more difficult motor tasks.

Suppression of GABA receptor functions has been reported to enhance motor learning consolidation and motor memory reconsolidation (Makkar et al., 2010). Similarly, Stagg et al. concluded that a reduction in GABAergic activity can enhance motor learning (Stagg et al., 2011), while Cardellicchio et al. reported that high GABA levels in the sensorimotor cortex inhibited motor learning (Cardellicchio and Borgomaneri, 2025). In accord with this reciprocal relationship between striatal GABAergic activity and motor learning, tVNS both enhanced early-phase motor learning and reduced striatal GABA+. A previous animal study identified the posterior dorsomedial striatum as a critical area for motor learning by associating sensory information with action outcomes (Reinhold, 2025). Human studies have also suggested that the STR is essential for early-phase motor learning (Janacsek et al., 2020; Stillman et al., 2013; Cataldi et al., 2022; Yin et al., 2009). reported that the caudate nucleus, a component of the STR, is involved in the early, rapid phase of motor learning while others have implicated the putamen (the other STR component) in later stages of learning. A functional MRI study (Choi et al., 2020) also revealed enhanced activity in the head of the caudate nucleus during the initial learning phase. Furthermore, recent studies have reported that noninvasive brain stimulation targeting the STR, such as transcranial temporal interference stimulation, can enhance striatal activity and facilitate motor learning (Ploumitsakou et al., 2025; Vassiliadis et al., 2024; Wessel et al., 2023). Given these similarities, it is conceivable that tVNS may serve as an alternative noninvasive neuromodulation approach to activate (disinhibit) the striatum and promote motor learning.

Furthermore, the DLPFC plays a central role in executive control, attention, and strategic aspects of motor learning (Friedman and Robbins, 2022; Barbey et al., 2013). Therefore, increased glutamatergic neurotransmission in the DLPFC may indicate enhanced cortical excitability and synaptic efficacy in motor learning.

The observed increase in DLPFC Glu, coupled with striatal disinhibition, likely amplified the excitatory cortical drive to the striatum, forming a corticostriatal loop critical for motor learning. Thus, the combination of reduced striatal GABA+ and increased prefrontal glutamatergic drive enhanced the information flow within this loop, facilitating more efficient coordination between top-down control and subcortical execution. Such coordinated modulation of the corticostriatal network is a probable mechanism for the accelerated early-phase motor learning observed in the present study.

**Figure 5:**
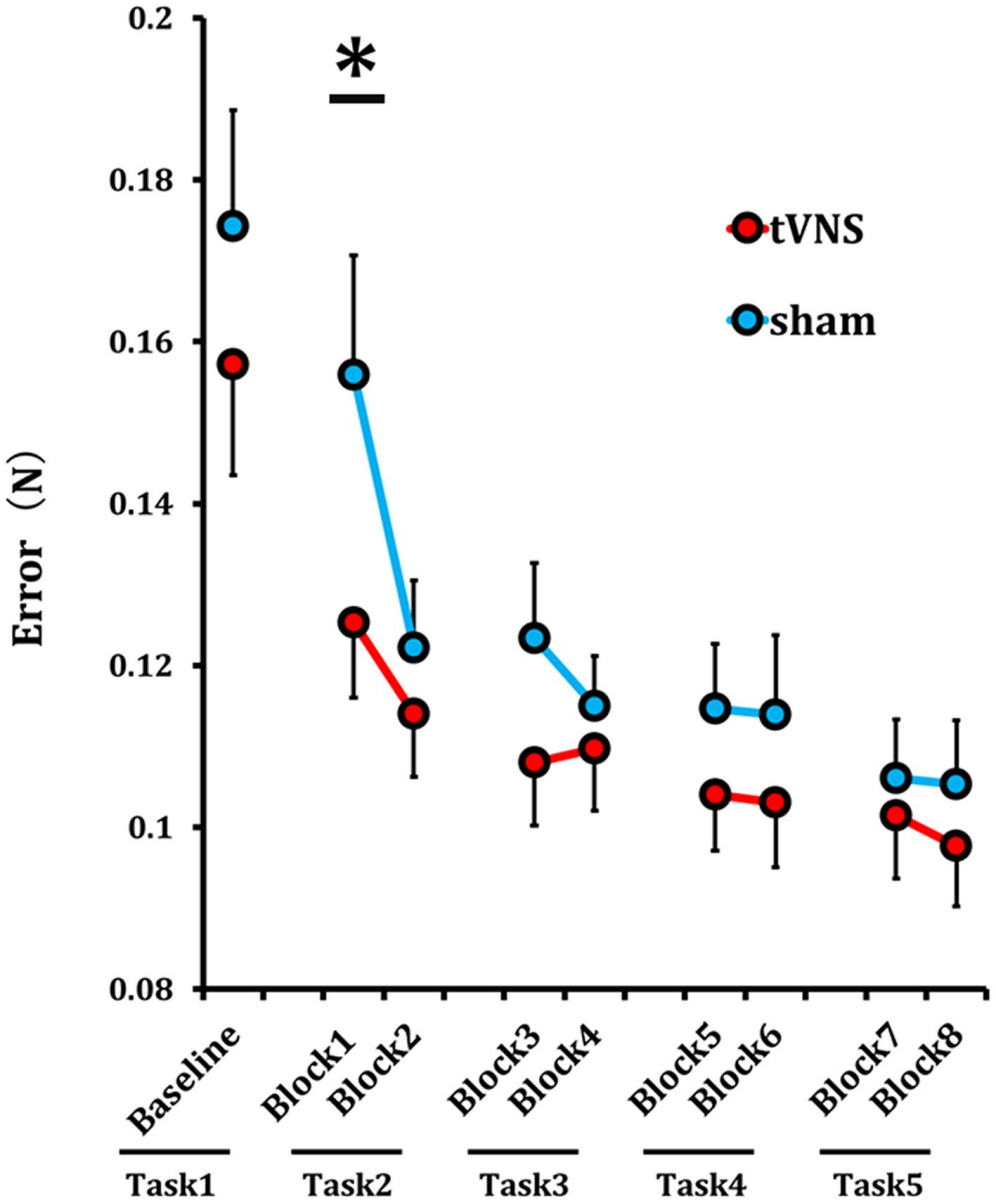
tVNS improved force-control motor task performance. Time course of the mean error during the force-control motor task. The red line represents the tVNS group and the blue line represents the sham group. Each task consisted of two blocks. Error values were averaged within each block and corresponding blocks compared between tVNS and sham groups. In block 1 (10 min post-stimulation), the tVNS group showed a significantly lower error value compared with the sham group (*p < 0.05). Error bars indicate the SD.

Although tVNS promotes early-phase motor learning, we cannot rule out the possibility that tVNS may also enhance the overall capacity for motor skill acquisition. In our study, performance improvements reached a plateau around Task 3, approximately 20 min after tVNS initiation (Figure 5). This ceiling effect may have limited our ability to detect further enhancements in motor performance. Employing a task with higher difficulty or extended duration may allow for a more accurate assessment of the potential effects of tVNS on the full capacity of motor learning. Future studies are guaranteed to explore this possibility.

### 4.3 Clinical implication

The observed reduction in striatal GABA+ levels and acceleration of early-phase motor learning suggest that tVNS may be a useful intervention for post-stroke rehabilitation as excessive tonic inhibition has been shown to hamper motor recovery. As a noninvasive and portable technique, tVNS holds practical advantages over other neuromodulation methods and may be more easily implemented in rehabilitation settings. Further research is needed to determine its efficacy in patient populations and to optimize stimulation protocols for clinical use.

### 4.4 Limitations

Our study has several limitations. First, Experiments 1 and 2 were conducted separately in different participant groups. Therefore, we could not establish a direct correlation between changes in striatal GABA+ content and motor learning performance. Second, it is technically challenging to measure GABA+ levels in multiple brain regions simultaneously using ^1^H-MRS. As a result, there was a time lag between scans for different brain regions, and transient changes induced by tVNS may have been missed. Moreover, we restricted our ^1^H-MRS measurements to the left hemisphere to minimize potential time-related biases, considering that each ^1^H-MRS acquisition required approximately 15 min per region. Although previous behavioral studies have shown that left-ear tVNS can influence right-hand motor performance, bilateral effects of tVNS on neurotransmitter modulation have not been clearly demonstrated (Toffa et al., 2020). Future studies should investigate possible bilateral neurotransmitter modulation in these regions. In addition, while ^1^H-MRS offers the advantage of non-invasive measurement, it does not distinguish between intracellular and extracellular GABA+ and thus among changes in synthesis, storage, and release into synaptic and presynaptic spaces. Third, while a previous study reported a lasting effect of tVNS on depression scores (Li et al., 2022), our study did not examine the after-effect of tVNS on GABAergic modulation and motor learning.

### 4.5 Future prospects

The present study demonstrated that a reduction in striatal GABA+ following tVNS may reflect engagement of subcortical pathways. Afferent vagal stimulation activates the nucleus tractus solitarius, which has strong anatomical and functional connectivity with the locus coeruleus (Yakunina et al., 2017; Hulsey et al., 2016; Owens et al., 2024; Mendonça-dos-Santos et al., 2024). Activating this pathway affects downstream subcortical regions, including the striatum, potentially inducing region-specific modulation of inhibitory neurotransmission (Proietti et al., 2025). Furthermore, striatal activity is also regulated by glutamatergic cortical inputs (Cataldi et al., 2022). The increase in DLPFC Glu levels indicates the potential modulation by tVNS may of cortico-striatal interactions. Together, these mechanisms may facilitate the region-dependent effects of tVNS on GABA+ levels.

Furthermore, we showed that tVNS can modulate striatal GABA+ and DLPFC Glu levels, thereby facilitating early motor learning. The observed decrease in striatal GABA+ may induce motor circuit plasticity, which has potential value in the rehabilitation of motor disorders, such as poststroke motor deficits or Parkinson’s disease. Similarly, increased DLPFC Glu may enhance cognitive control and learning, suggesting broader applicability to conditions involving executive dysfunction. Because baseline neurotransmitter levels and neural plasticity differ across individuals and patient populations, future studies should explore the personalization of stimulation parameters to optimize the clinical efficacy of tVNS.

However, in the present study, tVNS was applied during the execution of the motor task in Experiment 2, as described in previous studies (Chen et al., 2022). Animal studies have demonstrated that the timing of VNS delivery relative to motor learning critically influences plasticity and behavioral outcomes (Hays et al., 2014; Souza et al., 2022). Furthermore, although the relationship between stimulation timing and motor learning has also been investigated in human studies (Chen et al., 2022; Jongkees et al., 2018), the findings remain inconsistent. Notably, recent tVNS studies tend to report online effects, in which stimulation delivered during task performance facilitates learning, rather than offline effects observed after stimulation (St Pierre and Shinohara, 2023; Ravestein et al., 2025; Obata and Sekiguchi, 2025). Consistent with these findings, we believe that the effects observed in the present study were primarily driven by the online modulation of motor learning processes. Nevertheless, an optimal and consistent timing strategy for VNS delivery has not yet been established. Further studies are required to systematically investigate the interaction between stimulation timing and motor learning and performance.

To better clarify the relationship among tVNS, GABA+, and motor learning, further studies are needed to elucidate the underlying neurophysiological mechanisms. In this study, we were unable to simultaneously observe neurotransmitter modulation during stimulation or during a motor task. In future studies, we aim to conduct concurrent measurements, such as combining ^1^H-MRS assessments of GABA+ with motor learning tasks or stimulation. This approach would allow us to directly link tVNS-induced modulation of neurotransmitters to behavioral outcomes. Furthermore, examining individual differences in response to tVNS could provide insights for the development of potential clinical applications and the identification of individuals who may most benefit from this intervention.

## 5 Conclusion

Our study demosntrated that tVNS (1) contributes to a reduction in striatal GABA+ levels and increased DLPFC Glu levels in healthy adults and (2) facilitates early-phase motor learning. These findings support the use of tVNS as a noninvasive intervention for enhance motor learning in neurorehabilitation and the treatment of motor disorders.

## Data Availability

The raw data supporting the conclusions of this article will be made available by the authors, without undue reservation.
